# Hormonal regulation of floret closure of rice (*Oryza sativa*)

**DOI:** 10.1371/journal.pone.0198828

**Published:** 2018-06-07

**Authors:** Youming Huang, Xiaochun Zeng, Heping Cao

**Affiliations:** 1 Key Laboratory of Crop Physiology, Ecology and Genetic Breeding, Ministry of Education, Jiangxi Agricultural University, Nanchang, Jiangxi Province, China; 2 Department of Life Science and Environmental Resources, Yichun University, Yichun, Jiangxi Province, China; 3 U.S. Department of Agriculture, Agricultural Research Service, Southern Regional Research Center, Commodity Utilization Research Unit, New Orleans, Louisiana, United States of America; Louisiana State University College of Agriculture, UNITED STATES

## Abstract

Plant hormones play important roles in regulating every aspect of growth, development, and metabolism of plants. We are interested in understanding hormonal regulation of floret opening and closure in plants. This is a particularly important problem for hybrid rice because regulation of flowering time is vitally important in hybrid rice seed production. However, little was known about the effects of plant hormones on rice flowering. We have shown that jasmonate and methyl jasmonate play significant roles in promoting rice floret opening. In this study, we investigated the effects of auxins including indole-3-acidic acid (IAA), indole-3-butyric acid (IBA), 1-naphthalene-acetic acid (NAA), 2,4-dichlorophenoxy acetic acid (2,4-D) and 3,6-dichloro-2-methoxybenzoic acid (DIC) and abscisic acid (ABA) on floret closure of four fertile and three sterile varieties of rice. The results from field studies in three growing seasons in 2013–2015 showed that the percentages of closed florets were significantly lower in plants treated with IAA, IBA, 2,4-D, DIC and NAA and that the durations of floret opening were significantly longer in plants treated with the same auxins. The auxins exhibited time- and concentration-dependant effects on floret closure. ABA displayed opposite effects of auxins because it increased the percentages of floret closure and decreased the length of floret opening of rice varieties. The degree of auxin-inhibiting and ABA-promoting effects on floret closure was varied somewhat but not significantly different among the rice varieties. Endogenous IAA levels were the highest in florets collected shortly before opening followed by a sharp decline in florets with maximal angles of opening and a significant jump of IAA levels shortly after floret closure in both fertile and sterile rice plants. ABA levels showed an opposite trend in the same samples. Our results showed that auxins delayed but ABA promoted the closure of rice floret regardless of the varieties.

## Introduction

Plant hormones play important roles in regulating every aspect of growth, development and metabolism of plants. For example, brassinosteroids regulate rice lamina joint inclination due to uneven expansion of the cells in the joint [1]. Gibberellins (GAs) regulate the amount of starch and structural polysaccharides in maize endosperm suspension cells via regulating enzyme activities involved in carbohydrate metabolism [2]. Indole-3-acetic acid (IAA), GAs, cytokinins and abscisic acid (ABA) regulate sex differentiation of the flowers of Chinese Chinquapin (*Castanea henryi*) [3].

We are interested in understanding hormonal regulation of floret opening and closure in plants. This is a particularly important problem for hybrid rice because regulation of flowering time is vitally important in hybrid rice seed production. Rice (*Oryza sativa*) flowering is a developmental process involved in multiple steps including inflorescence initiation, development of the flower structure, panicle extrusion (heading) and flower opening (anthesis). In this study, rice flowering is narrowly defined as anthesis for simplicity of description and refers to the opening and closing of the floret which takes 6–10 days for the tens-hundreds of florets within the same panicle to complete flowering [4]. Rice flowering time occurs at a certain time of the day and varies among different varieties and under different weather conditions. Fertile rice varieties normally start flowering at 9–10 am of the day and last for 1–2 h. However, the flowers of sterile rice varieties open later, take longer time to close and last 4–8 h [5,6]. The extended flowering times for the sterile rice expose them to a number of disadvantages, such as abnormal development of ovaries [7], susceptible to rice kernel smut [8–10] and increasing the percentage of glume-gaping grains up to 12–61% [11,12]. These disadvantages hinder the production of hybrid rice because they greatly affect the quality and quantity of hybrid seeds. However, little was known about the effects of plant hormones on rice flowering.

Plant hormone jasmonates including jasmonate (JA) and its methyl ester, methyl jasmonate (MeJA) are important regulators of plant reproductive development [13,14]. We were the first to demonstrate that JA and MeJA played important roles in the promotion of rice and sorghum flower opening [15,16]. This discovery led to new technology development for regulating flowering time during hybrid rice seed production [17]. However, much less was known about the regulation of floret closure by plant hormones. In this study, we investigated the regulation of floret closure of fertile rice and sterile rice by auxins including IAA, indole-3-butyric acid (IBA), 1-naphthalene-acetic acid (NAA), 2,4-dichlorophenoxy acetic acid (2,4-D) and 3,6-dichloro-2-methoxybenzoic acid (Dicamba or DIC) and ABA. Field studies in three growing seasons between 2013 and 2015 showed that auxins delayed but ABA promoted the closure of rice floret regardless of the varieties.

## Materials and methods

### Ethics statement

No specific permits were required from collecting the samples because rice plants were public-owned and the field studies did not involve protected species. No human or animals were involved in this study.

### Plants

Rice (*Oryza sativa*) varieties used in the experiments include fertile lines (Huang-yue-zhan, Jin-you-gui-99, Ping-hui-141, Jia-zao-211) and sterile lines (Wu-feng-A, Zhong-9A, Bo-you-A). These rice varieties included indica rice (Huang-yue-zhan, Ping-hui-141, Wu-feng-A, Zhong-9A), indica hybrid rice (Jin-you-gui-99), japonica rice (Bo-you-A) and japonica hybrid rice (Jia-zao-211). All plants but Jia-zao-211 were planted on the experimental field at Yichun University and Jia-zao-211 was planted at the Science and Technology Park of Jiangxi Agricultural University. The plants were grown under natural climate conditions. The rice plants grown in Yichun City, Jiangxi Province has the following geographical coordinates (27°33′—29°06′N, 113°54′—116°27′E). The annual average temperature, sunlight, and water fall was 17.2°C, 1737.1 h, and 1680.2 mm, respectively. Field experiments were conducted in three growing seasons from May to September in 2013–2015 and the heading to flowering period was approximately one week in July of each year. This investigation was a pioneer study of the regulation of floret closure in rice by auxins and ABA. Our initial emphasis was to explore as many varieties as possible to confirm our original observation on the hormonal regulation of rice floret closure. There were some variations of the effects of hormones on rice floret closure but no significant difference was observed among the different varieties.

### Chemicals

Plant hormones and their synthetic analogs used in the experiments were purchased from Sigma. They were abscisic acid (ABA, Sigma A1049), indole-3-acidic acid (IAA, Sigma I2886), indole-3-butyric acid (IBA, Sigma I5386), 1-naphthalene-acetic acid (NAA, Sigma N0640), 2,4-dichlorophenoxy acetic acid (2,4-D, Sigma D6679) and 3,6-dichloro-2-methoxybenzoic acid (Dicamba or DIC, Sigma D5417). Plant hormones were dissolved in 10 volumes of ethanol according to manufacturer’s instructions and then diluted with water to the desired concentrations before uses. The control received the same amount of ethanol equal to those in dissolving the chemicals.

### Chemical treatment

ABA and NAA were used at five concentrations (50, 100, 200, 400 and 800 mg/L). IAA, IBA, 2,4-D and DIC were used at four concentrations (50, 100, 200 and 400 mg/L). ABA concentrations at 50, 100, 200, 400 and 800 mg/L corresponded to ≈ 0.19, 0.38, 0.76, 1.52, and 3.0 mM, respectively. NAA concentrations at 50, 100, 200, 400 and 800 mg/L corresponded to ≈ 0.27, 0.54, 1.1, 2.2, and 4.4 mM, respectively. IAA concentrations at 50, 100, 200, and 400 mg/L corresponded to ≈ 0.29, 0.57, 1.14, and 2.28 mM, respectively. IBA concentrations at 50, 100, 200, and 400 mg/L corresponded to ≈ 0.25, 0.5, 1.0 and 1.97 mM, respectively. 2,4-D concentrations at 50, 100, 200, and 400 mg/L corresponded to ≈ 0.23, 0.45,0.9, and 1.8mM, respectively. DIC concentrations at 50, 100, 200, and 400 mg/L corresponded to ≈ 0.23, 0.45, 0.9, and 1.8mM, respectively. The controls contained all other solvents without growth regulators. Rice plants with similar growth and development stages were sprayed the chemicals at 500 mL/5m^2^ approximately one hour before floret opening under clear sky conditions. The treatments were not performed in the same day among the different varieties. Instead, they were treated according to flower development. The selection of treatment time was based on the development of anther and the top floret opening the day before. Specifically, treatment “one hour before floret opening” was determined by field observation of flower development several days prior to the treatment based on the height of anther in the flower and the opening time of the top floret the day before. The concentrations of plant hormones used in this study at the μM-mM levels were based on previous studies for cereals [1,2,15,18]. No visible phenotypic change was observed in rice plants treated with these hormones during the course of study.

### Dynamics of floret closure

Rice plants with several florets on the upper part of the plant flowered the day before the treatment and the middle part of the plant judged to be flowering massively were selected for tests. The number of closed florets was counted every 30 min after the first floret closure until the last floret was closed. Three plants were selected for each observation. Each treatment was repeated three times (three panicles). The % of closed florets in the Figs represents the number of closed floret in the selected time point/total closed florets of the day. Zero min started at the first closed floret observed. The closed florets were examined every 30-min during the day. The length of floret closure in the Figs represents the mean of 3 replications with each measurement containing 50 florets.

### Measurement of endogenous levels of plant hormones

Three stages of florets were collected: shortly prior to opening (before opening), maximal opening, and just after closure (after closure). The dissected florets were frozen in liquid nitrogen immediately and stored in an ultralow freezer. Each sample weighed 2 g and each treatment was repeated three times. The frozen tissue (0.9–1.1 g) was ground into a fine powder with a pre-cold mortar and pestle for 5 min in 2 mL of 80% ethanol. The powder was transferred into a 10 mL centrifuge tube. The mortar was washed with 2 mL of 80% ethanol twice and pooled in the same centrifuge tube which was left for extraction at 4°C for 4 h or overnight followed by centrifugation at 4,000 rpm for 15 min. The residue was extracted with 1 mL of 80% ethanol at 4°C for 12 h and centrifuged again. The combined supernatant was purified with C_18_ column, dried with N_2_ and stored at -20°C. Endogenous levels of IAA and ABA were determined by enzyme-linked immunosorbent assays (ELISA) using a kit provided by the Chemical Regulation Laboratory of the China Agricultural University [19,20]. The measurement was done with Model 680 (Bio-Rad Laboratories) or ELX 800 (CHINETEK Company). The concentration and content of hormones were calculated using the database provided by the Chemical Regulation Laboratory of the China Agricultural University. Three samples were determined and the mean was used for statistics.

### Data analysis

Data analysis was similar to what we used previously [2,21]. The data in the Figs and tables were the means and standard deviations of three biological sets of data with each biological set came from three technical measurements. Mean values within table columns were analyzed with Duncan new multiple range test [22]. Values in a column with different letters indicate a significant different difference among the floret stages. Uppercase and lowercase letters represent significant differences between floret stages at ρ = 0.01 and ρ = 0.05, respectively.

## Results

### Auxins delayed floret closure of fertile rice varieties

**Fig 1:**
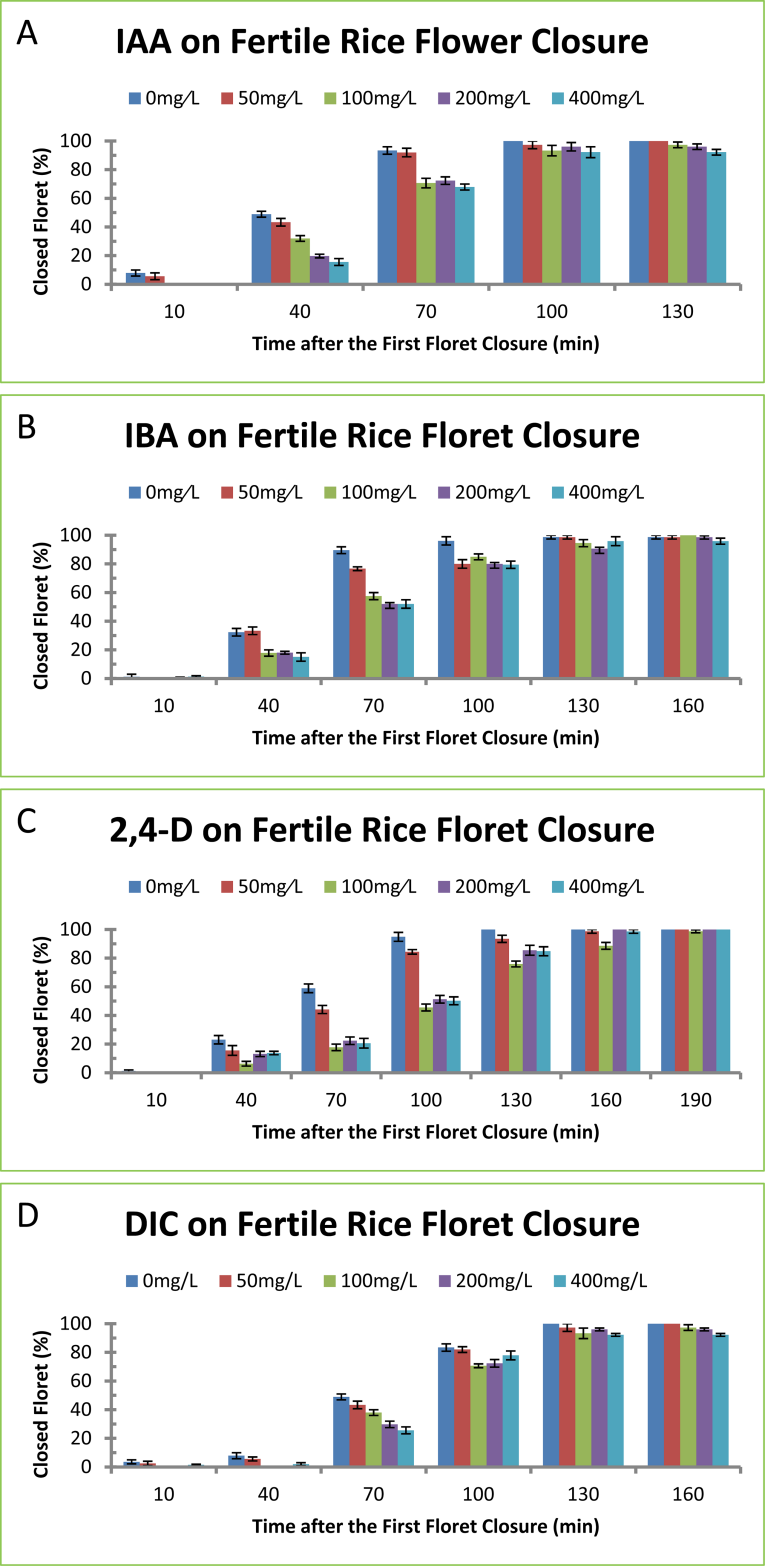
Effect of auxins on floret closing in fertile rice variety Jia-zao-211. (A) IAA. The concentrations of IAA at 50, 100, 200 and 400 mg/L correspond to 0.29, 0.57, 1.14 and 2.28 mM, respectively. (B) IBA. The concentrations of IBA at 50, 100, 200 and 400 mg/L correspond to 0.25, 0.49, 0.98 and 1.97 mM, respectively. (C) 2,4-D. The concentrations of 2,4-D at 50, 100, 200 and 400 mg/L correspond to 0.23, 0.45, 0.90 and 1.81 mM, respectively. (D) DIC. The concentrations of DIC at 50, 100, 200 and 400 mg/L correspond to 0.23, 0.45, 0.90 and 1.81 mM, respectively. The data in the Fig were the means and standard deviations of three independent samples.

**Fig 2:**
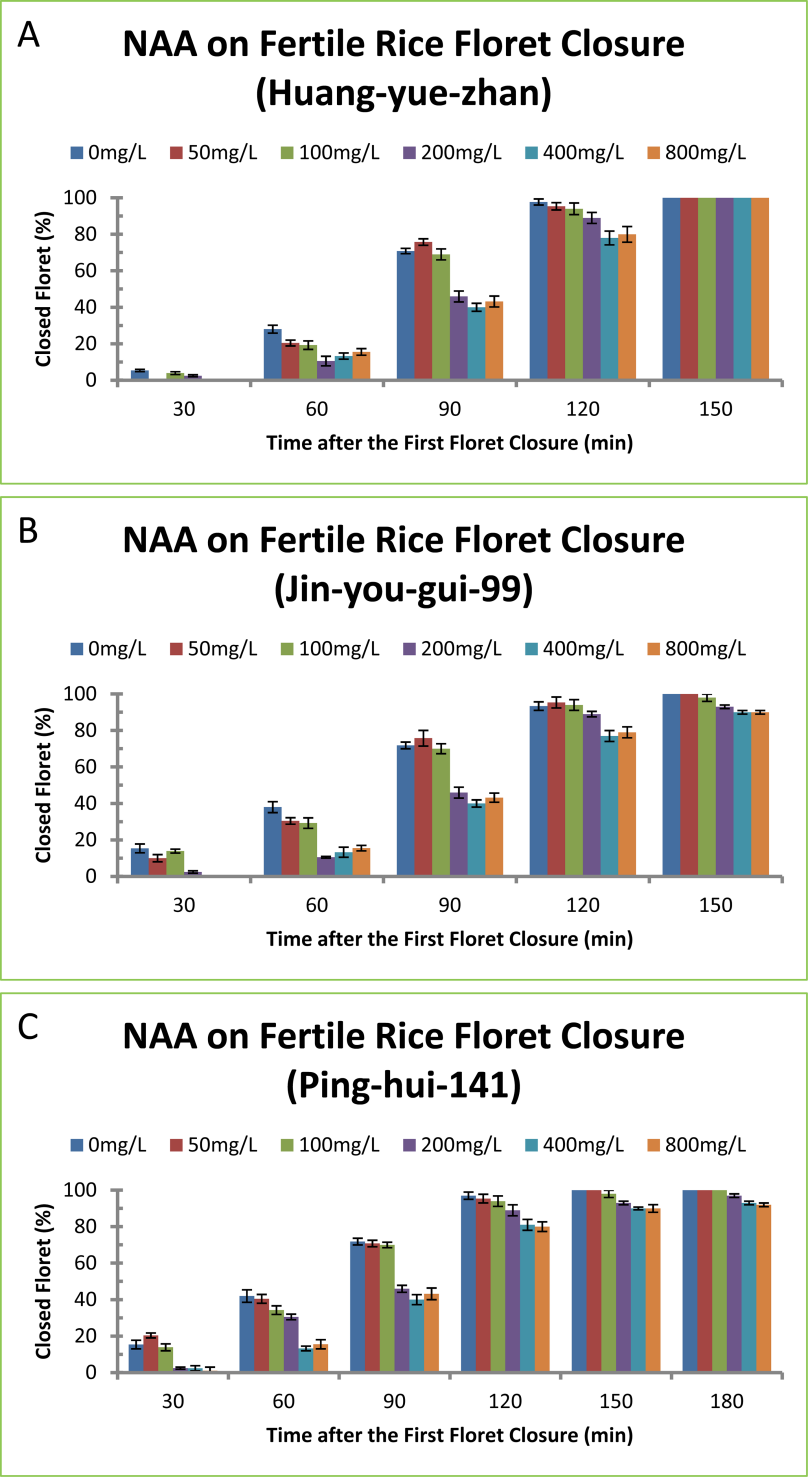
Effect of NAA on floret closing in three fertile rice varieties. (A) Huang-yue-zhan, (B) Jin-you-gui-99, (C) Ping-hui-141. The concentrations of NAA at 50, 100, 200, 400 and 800 mg/L correspond to 0.27, 0.54, 1.07, 2.15 and 4.30 mM, respectively. The data in the Fig were the means and standard deviations of three independent samples.

IAA and IBA are naturally occurring auxins in plants. 2,4-D and DIC are synthetic auxins widely used in agriculture. These auxins were initially tested for their effects on floret closure of rice variety Jia-zao-211. Rice plants were selected for the tests based on the facts that those plants had several florets on the upper part of the plants already flowered and that the middle part of the plants were judged to be flowered massively the day before the treatment. The number of closed florets was counted every 30 min after the first floret closure until the last floret was closed. The length of floret closure was recorded with 50 florets per plant. The percentages of closed florets were clearly lower in plants treated with IAA (Fig 1A), IBA (Fig 1B), 2,4-D (Fig 1C) and DIC (Fig 1D) compared to the control, especially 40- and 70-min after the first floret closure. The durations of floret opening were significantly longer in plants treated with IAA, IBA, 2,4-D and DIC compared to the control (Table 1). The auxins on floret closure exhibited time- and concentration-dependant effects. The longer time after auxin treatment resulted in higher percentages of closed florets (Fig 1). The higher concentrations of auxin treatment resulted in longer time of floret opening (Table 1). Auxins at 400 mg/L did not result in more significant effects on delaying floret closure than those at 200 mg/L as shown by the trend of changes (Table 1). IAA treatment at 200 and 400 mg/L delayed floret closure for 10 and 11 min (Table 1), and the percentages of closed florets were 14 and 15% less than the control plant, respectively (Fig 1A). IBA treatment at 200 and 400 mg/L delayed floret closure for 9 and 10 min (Table 1), and the percentages of closed florets were 13 and 14% less than the control plant, respectively (Fig 1B). 2,4-D treatment at 200 and 400 mg/L delayed floret closure for 9 and 10 min (Table 1), and the percentages of closed florets were 12 and 14% less than the control plant, respectively (Fig 1C). DIC treatment at 200 and 400 mg/L delayed floret closure for 13 and 14 min (Table 1), and the percentages of closed florets were 18 and 20% less than the control plant, respectively (Fig 1D).

**Table 1:** Auxin regulation of the duration of floret opening in fertile rice.

Concentration (mg/L) (mM)	Duration of Floret Opening (min)			
	IAA	IBA	2,4-D	DIC
0	71.62 ^B^	69.58 ^c,B^	70.50 ^b,C^	69.50 ^B^
50 (0.29)	70.44 ^B^	70.48 ^c,B^	72.15 ^b,C^	72.30 ^B^
100 (0.57)	73.50 ^B^	73.22 ^bc,AB^	72.38 ^b,BC^	73.66 ^B^
200 (1.14)	81.78 ^A^	78.50 ^ab,A^	79.30 ^a,AB^	82.12 ^A^
400 (2.28)	82.29 ^A^	79.50 ^a,A^	80.69 ^a,A^	83.25 ^A^

Mean values within a column were analyzed with Duncan new multiple range test. Values in a column with different letters indicate a significant difference among the treatments. Uppercase and lowercase letters represent significant differences at ρ = 0.01 and ρ = 0.05, respectively.

To confirm this effect of auxins on delaying floret closure observed in one rice variety, another widely used auxin, NAA, was selected to test on three different rice varieties (Fig 2). NAA displayed similar effects on delaying the percentages of floret closure of rice varieties Huang-yue-zhan (Fig 2A), Jin-you-gui-99 (Fig 2B) and Ping-hui-141 (Fig 2C) and increasing the length of floret opening (Table 2). Like other auxins, NAA on floret closure also exhibited time- and concentration-dependant effects. Longer time after NAA treatment resulted in higher percentages of closed florets (Fig 2). Higher concentrations of NAA treatment resulted in longer time of floret opening (Table 2). NAA treatment of rice variety Huang-yue-zhan at 100, 200, 400 and 800 mg/L delayed floret closure for 7, 14, 19 and 19 min (Table 2), and the percentages of closed florets were 9, 18, 25 and 25% less than the control plant, respectively (Fig 2A). NAA treatment of rice variety Jin-you-gui-99 at 200, 400 and 800 mg/L delayed floret closure for 12, 19 and 18 min (Table 2), and the percentages of closed florets were 16, 25 and 24% less than the control plant, respectively (Fig 2B). NAA treatment of rice variety Ping-hui-141 at 200, 400 and 800 mg/L delayed floret closure for 17, 22 and 23 min (Table 2), and the percentages of closed florets were 24, 31 and 32% less than the control plant, respectively (Fig 2C). NAA at 400 mg/L appeared to have maximal effect on floret closure because NAA at 800 mg/L did not result in further delaying of floret closure (Fig 2).

**Table 2:** NAA regulation of the duration of floret opening in fertile rice.

Concentration (mg/L) (mM)	Duration of Floret Opening (min)		
	Huang-yue-zhan	Jin-you-gui-99	Ping-hui-141
0	76.33 ^d,B^	76.37 ^c,B^	71.30 ^c,B^
50 (0.27)	80.53 ^d,B^	81.82 ^c,B^	73.27 ^c,B^
100 (0.54)	90.35 ^b,AB^	85.02 ^bc,B^	75.06 ^c,B^
200 (1.1)	95.30 ^a,A^	88.38 ^b,AB^	93.16 ^ab,A^
400 (2.2)	95.55 ^a,A^	94.95 ^a,A^	94.05 ^a,A^

Mean values within a column were analyzed with Duncan new multiple range test. Values in a column with different letters indicate a significant difference among the treatments. Uppercase and lowercase letters represent significant differences at ρ = 0.01 and ρ = 0.05, respectively.

### Abscisic acid promoted floret closure of fertile rice varieties

**Fig 3:**
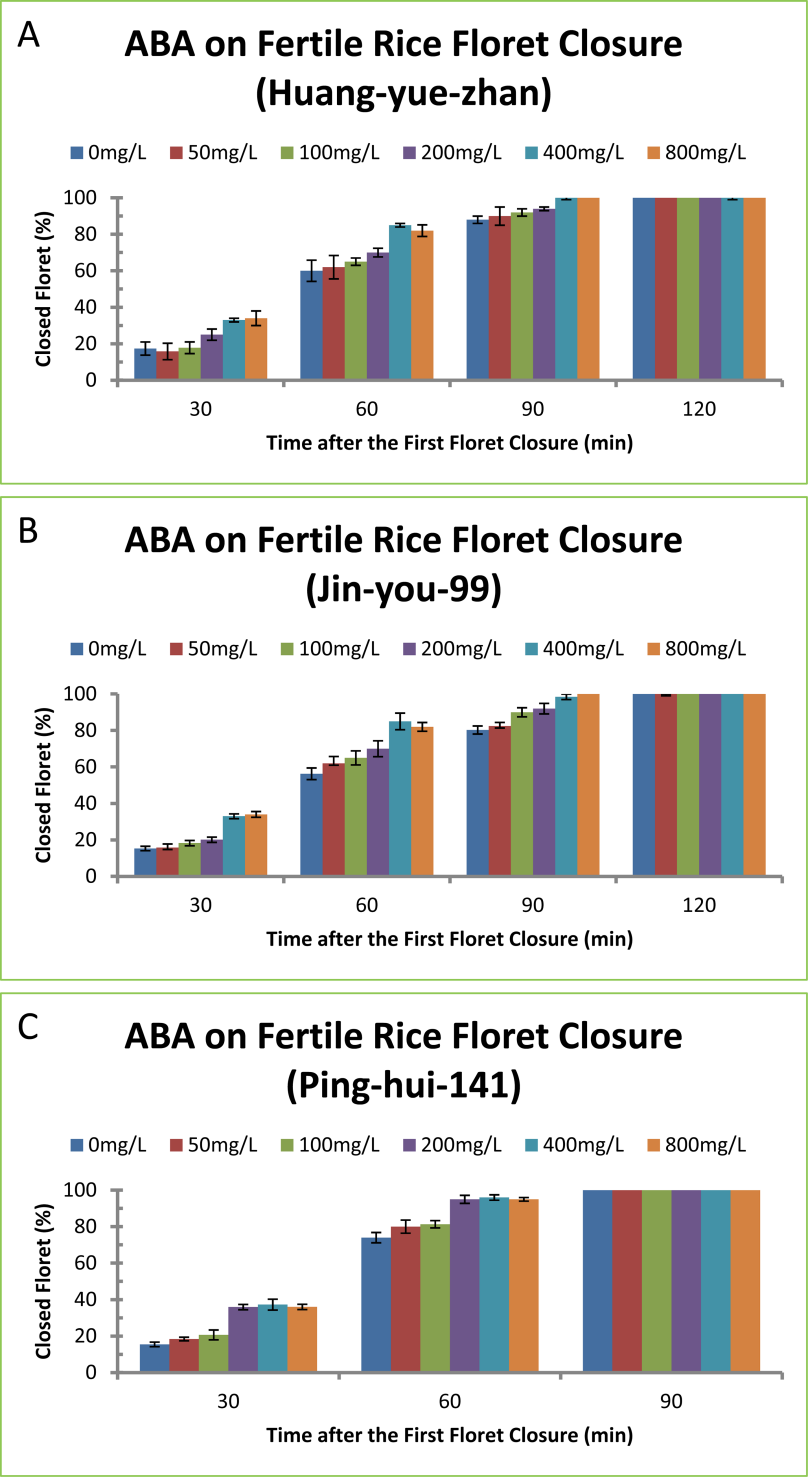
Effect of ABA on floret closing in three fertile rice varieties. (A) Huang-yue-zhan, (B) Jin-you-gui-99, (C) Ping-hui-141. The concentrations of ABA at 50, 100, 200, 400 and 800 mg/L correspond to 0.19, 0.38, 0.76, 1.51 and 3.30 mM, respectively. The data in the Fig were the means and standard deviations of three independent samples.

ABA is well-known to promote stomata closure in plant leaves. We tested if ABA also accelerated floret closure during rice flower development. Our results showed that ABA displayed opposite effects of auxins because it increased the percentages of closed florets and decreased the length of floret opening of rice varieties Huang-yue-zhan, Jin-you-gui-99 and Ping-hui-141 (Fig 3 and Table 3). ABA on floret closure also exhibited time- and concentration-dependant effects. ABA treatment for longer time resulted in higher percentages of closed florets (Fig 3), and higher concentrations of ABA treatment resulted in shorter time of floret opening (Table 3). ABA exhibited minor difference among different rice varieties. ABA treatment of rice variety Huang-yue-zhan at 200, 400 and 800 mg/L promoted floret closure by 11, 16 and 16 min (Table 3), and the percentages of closed florets in ABA-treated plants were 14, 20 and 20% more than the control plant, respectively (Fig 3A). ABA treatment of rice variety Jin-you-gui-99 at 100, 200, 400 and 800 mg/L promoted floret closure by 13, 20, 27 and 25 min (Table 3), and the percentages of closed florets in ABA-treated plants were 16, 25, 34 and 31% more than the control plant, respectively (Fig 3B). ABA treatment of rice variety Ping-hui-141 at 100, 200, 400 and 800 mg/L promoted floret closure by 7, 10, 19 and 17 min (Table 3), and the percentages of closed florets in ABA-treated plants were 9, 13, 25 and 23% more than the control plant, respectively (Fig 3C). ABA at 400 mg/L had the maximal effect on floret closure because ABA at 800 mg/L did not result in further delaying of floret closure (Fig 3).

**Table 3:** ABA regulation of the duration of floret opening in fertile rice.

Concentration (mg/L) (mM)	Duration of Floret Opening (min)		
	Huang-yue-zhan	Jin-you-gui-99	Ping-hui-141
0	81.23 ^a,A^	80.29 ^a,A^	76.05 ^a,A^
50 (0.19)	80.47 ^a,A^	80.45 ^a,A^	73.42 ^ab,AB^
100 (0.38)	77.77 ^a,AB^	67.07 ^b,B^	69.17 ^bc,AB^
200 (0.76)	70.15 ^b,B^	60.15 ^c,BC^	66.25 ^b,B^
400 (1.52)	65.38 ^b,B^	53.08 ^c,C^	56.83 ^c,D^
800 (3.0)	65.25 ^b,B^	55.65 ^cd,C^	58.64 ^c,D^

Mean values within a column were analyzed with Duncan new multiple range test. Values in a column with different letters indicate a significant difference among the treatments. Uppercase and lowercase letters represent significant differences at ρ = 0.01 and ρ = 0.05, respectively.

### Auxin delayed floret closure of sterile rice varieties

**Fig 4:**
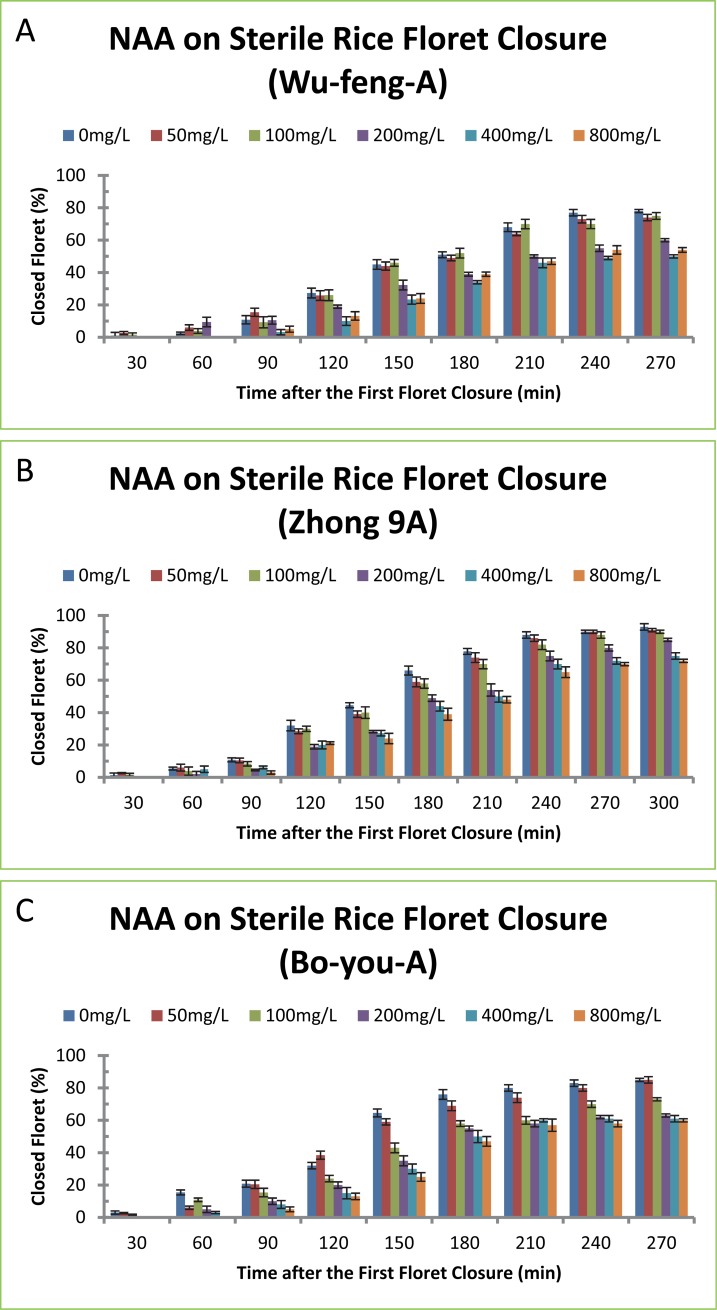
Effect of NAA on floret closing in three sterile rice varieties. (A) Wu-feng-A, (B) Zhong-9A, (C) Bo-you-A. The concentrations of NAA at 50, 100, 200, 400 and 800 mg/L correspond to 0.27, 0.54, 1.07, 2.15 and 4.30 mM, respectively. The data in the Fig were the means and standard deviations of three independent samples.

We tested whether auxins had similar effects on delaying floret closure in three sterile rice varieties. NAA was chosen for this experiment. The reason NAA was selected as the main auxin in this experiment was because NAA is widely used in agriculture and therefore the results from NAA might have more practical uses. NAA displayed similar effects on delaying the percentages of closed florets and increasing the length of floret opening of rice varieties Wu-feng-A, Zhong-9A and Bo-you-A (Fig 4 and Table 4). Similar to fertile rice varieties, NAA on floret closure exhibited time- and concentration-dependant effects on sterile rice varieties, i.e., longer NAA treatment resulted in higher percentages of closed florets (Fig 4) and higher concentrations of NAA treatment resulted in longer time of floret opening (Table 4). The effects of NAA on floret closure were also varied slightly among different rice varieties. NAA treatment of rice variety Wu-feng-A at 200, 400 and 800 mg/L delayed floret closure for 21, 45 and 51 min (Table 4), and the percentages of closed florets in NAA-treatment plants were 13, 28 and 32% less than the control plant, respectively (Fig 4A). NAA treatment of rice variety Zhong-9A at 200, 400 and 800 mg/L delayed floret closure for 71, 105 and 102 min (Table 4), and the percentages of closed florets in NAA-treatment plants were 34, 50 and 49% less than the control plant, respectively (Fig 4B). NAA treatment of rice variety Bo-you-A at 100, 200, 400 and 800 mg/L delayed floret closure for 13, 15, 38 and 35 min (Table 4), and the percentages of closed florets in NAA-treatment plants were 9, 11, 26 and 24% less than the control plant, respectively (Fig 4C). NAA at 400 mg/L and 800 mg/L had similar effect on floret closure (Fig 4).

**Table 4:** NAA regulation of the duration of floret opening in sterile rice.

Concentration (mg/L) (mM)	Duration of Floret Opening (min)		
	Wu-feng-A	Zhong-9A	Bo-you-A
0	159.00 ^c,C^	209.20 ^c,C^	145.24 ^c,B^
50 (0.27)	160.22 ^c,C^	206.27 ^c,C^	147.67 ^c,B^
100 (0.54)	175.03 ^bc,BC^	215.29 ^c,C^	157.91 ^b,B^
200 (1.1)	180.44 ^b,B^	280.48 ^b,B^	160.58 ^b,B^
400 (2.2)	203.76 ^a,AB^	313.76 ^a,A^	183.66 ^a,A^
800 (4.4)	210.02 ^a,A^	310.79 ^a,AB^	180.09 ^a,A^

Mean values within a column were analyzed with Duncan new multiple range test. Values in a column with different letters indicate a significant difference among the treatments. Uppercase and lowercase letters represent significant differences at ρ = 0.01 and ρ = 0.05, respectively.

### Abscisic acid promoted floret closure of sterile rice varieties

**Fig 5:**
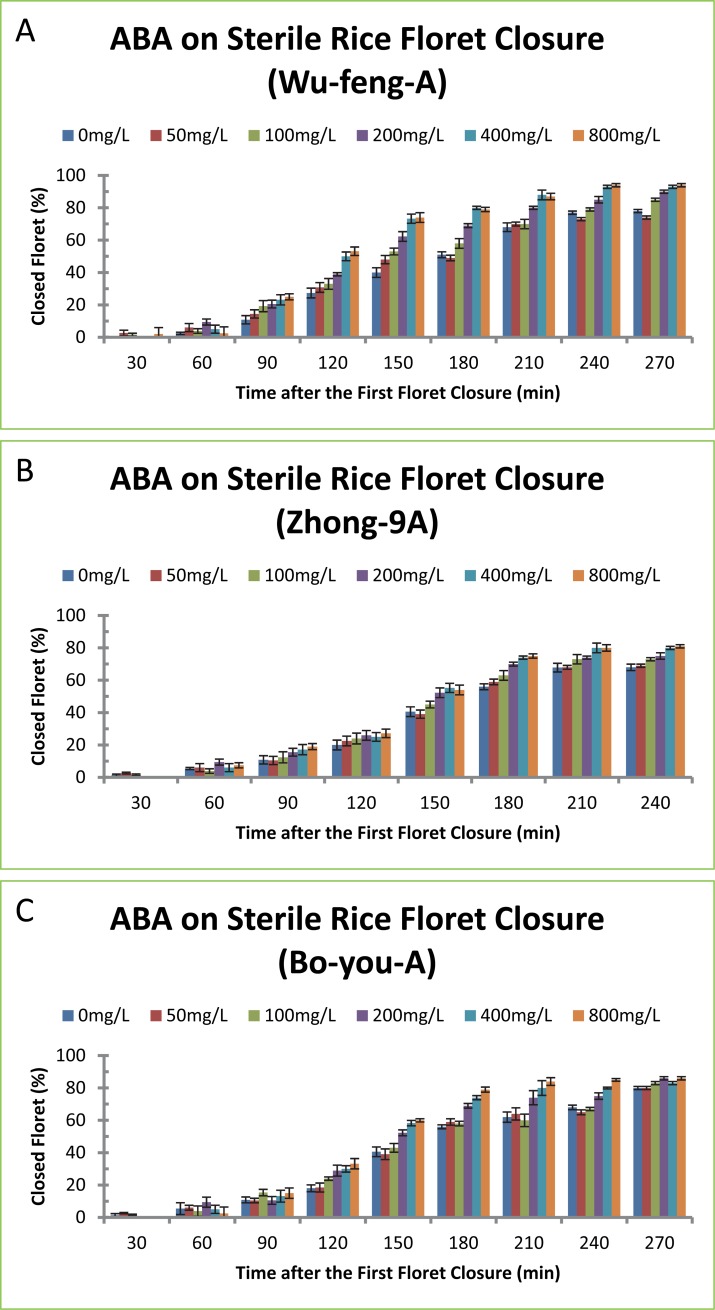
Effect of ABA on floret closing in three sterile rice varieties. (A) Wu-feng-A, (B) Zhong-9A, (C) Bo-you-A. The concentrations of ABA at 50, 100, 200, 400 and 800 mg/L correspond to 0.19, 0.38, 0.76, 1.51 and 3.30 mM, respectively. The data in the Fig were the means and standard deviations of three independent samples.

We tested whether ABA had similar effects on promoting floret closure in three sterile rice varieties. Like fertile rice varieties, ABA treatment increased the percentages of closed florets and decreased the length of floret opening of sterile rice varieties Wu-feng-A, Zhong-9A and Bo-you-A (Fig 5 and Table 5). These ABA effects on floret closure were also time- and concentration-dependant. ABA treatment for longer time resulted in lower percentages of closed florets (Fig 5) and ABA treatment with higher concentrations resulted in shorter time of floret opening (Table 5). ABA exhibited minor difference among different rice varieties. ABA treatment of rice variety Wu-feng-A at 200, 400 and 800 mg/L promoted floret closure by 21, 35 and 43 min (Table 5), and the percentages of closed florets in ABA-treated plants were 13, 22 and 26% more than the control plant, respectively (Fig 5A). ABA treatment of rice variety Zhong-9A at 100, 200, 400 and 800 mg/L promoted floret closure by 17, 32 and 37 min (Table 5), and the percentages of closed florets in ABA-treated plants were 8, 15 and 17% more than the control plant, respectively (Fig 5B). ABA treatment of rice variety Bo-you-A at 200, 400 and 800 mg/L promoted floret closure by 17, 22 and 26 min (Table 5), and the percentages of closed florets in ABA-treated plants were 11, 15 and 18% more than the control plant, respectively (Fig 5C). ABA treatment at 400 mg/L exhibited the maximal effect on floret closure because higher concentration of ABA at 800 mg/L did not result in further delaying of floret closure (Fig 5).

**Table 5:** NAA regulation of the duration of floret opening in sterile rice.

Concentration (mg/L) (mM)	Duration of Floret Opening (min)		
	Wu-feng-A	Zhong-9A	Bo-you-A
0	161.25 ^a,A^	217.29 ^a,A^	147.21 ^ab,AB^
50 (0.27)	165.32 ^a,A^	210.42 ^cb,AB^	153.22 ^a,A^
100 (0.54)	168.07 ^a,A^	203.73 ^b,AB^	138.63 ^b,BC^
200 (1.1)	140.55 ^b,B^	200.05 ^b,B^	130.65 ^c,BC^
400 (2.2)	125.80 ^c,C^	180.25 ^c,C^	125.05 ^cd,C^
800 (4.4)	118.60 ^c,C^	180.02 ^c,C^	120.92 ^d,C^

Mean values within a column were analyzed with Duncan new multiple range test. Values in a column with different letters indicate a significant difference among the treatments. Uppercase and lowercase letters represent significant differences at ρ = 0.01 and ρ = 0.05, respectively.

### Endogenous IAA and ABA levels before and after floret opening

ELISA was used to measure the concentrations of endogenous IAA and ABA levels in three stages of rice florets: shortly prior to opening, the maximal angle of opening and shortly after closure. Florets from Jin-you-gui-99 (fertile rice) and Zhong-9A (sterile rice) were collected for hormone analyses. IAA levels were the highest in florets collected shortly before opening followed by a sharp decline in florets with maximal angles of opening and a significant jump of IAA levels shortly after floret closure in both fertile and sterile rice plants (Table 6). IAA levels in florets from fertile rice variety (Jin-you-gui-99) and sterile rice variety (Zhong-9A) were 48 and 49 ng/g of fresh weight in florets before opening, respectively (Table 6), but IAA levels in florets with maximal opening angles decreased to 40 (Jin-you-gui-99) and 33 (Zhong-9A) ng/g of fresh weight, respectively (Table 6), a decline of 17 and 33%, respectively. Shortly after floret closure, IAA levels in these florets from fertile and sterile rice plants increased significantly to 46 (Jin-you-gui-99) and 38 (Zhong-9A) ng/g of fresh weight, respectively (Table 6).

**Table 6:** Endogenous levels of IAA and ABA in rice floret (ng/g.FW).

Floret Stage	IAA		ABA	
	Fertile Rice	Sterile Rice	Fertile Rice	Sterile Rice
Before opening	48.02 ^a^	49.29 ^a,A^	77.27 ^B^	74.78 ^b,AB^
Maximal opening	40.65 ^b^	33.21 ^b,B^	94.21 ^A^	85.91 ^a,A^
After closure	46.34 ^ab^	37.72 ^b,AB^	74.45 ^B^	72.63 ^b,B^

Mean values within a column were analyzed with Duncan new multiple range test. Values in a column with different letters indicate a significant difference among the floret stages. Uppercase and lowercase letters represent significant differences at ρ = 0.01 and ρ = 0.05, respectively.

On the contrary to IAA levels, ABA levels were the lowest in florets collected shortly before opening followed by a sharp increase in florets with maximal angles of opening and a significant decrease of ABA levels shortly after floret closure in both fertile and sterile rice plants (Table 6). ABA levels in florets from fertile rice (Jin-you-gui-99) and sterile rice (Zhong-9A) were 77 and 75 ng/g of fresh weight in florets before opening, respectively (Table 6), but ABA levels in florets with maximal opening angles increased to 94 (Jin-you-gui-99) and 86 (Zhong-9A) ng/g of fresh weight, an increase of 22 and 15%, respectively (Table 6). Shortly after floret closure, ABA levels in these florets from fertile and sterile rice plants decreased significantly to 74 (Jin-you-gui-99) and 73 (Zhong-9A) ng/g of fresh weight, respectively (Table 6).

## Discussion

This study revealed that plant hormones ABA and auxins (both endogenous and synthetic compounds) promoted and delayed floret closing of rice, respectively. To our knowledge, this is the first report related to the effects of the five classical plant hormones on rice floret closure. In previous studies, treatment with plant hormone MeJA delayed wheat floret closure for 42–70 min [23]. Salicylic acid (SA), another plant hormone, doubled the percentage of glume-gaping grains of sterile wheat after the floret opening was induced by MeJA [24]. ABA was shown to promote floret opening in *Ipomoea nil* and IAA was shown to inhibit floret opening in *Pharbitis nil* [25,26]. We performed similar experiments but we did not observe significant effect of ABA and IAA on floret opening in rice (data not shown). These suggest that the mechanisms of hormonal regulated floret opening in *Ipomoea nil* and *Pharbitis nil* are different from those in rice and wheat. The different responses of rice and the other species to the same hormonal regulation might be due to the different structures of florets in these two groups of species, in which rice floret opening is caused by the expansion of basal lodicules whereas the other two species do not have similar floral structure. Alternatively, although we thought unlikely, the different observation in these two plant species was due to much higher concentrations of ABA and auxins used in our studies than those used in the previous study.

Our study also showed that the endogenous levels of IAA were significantly decreased but those of ABA were increased in florets with maximal openings, although the levels of these hormones in fertile rice were much higher than those in sterile rice. These findings are similar to those of floret closing effects caused by exogenously applied auxins and ABA. Uchiumi and Okamoto [19] reported that IAA levels went up after pollination in rice fruit. They further showed that IAA levels in ovaries were lower than those in rachilla-pedicel before anthesis, became similar in the two tissues 3–6 h after flowering, and turned higher one day after flowering. Our results showed that IAA levels went up from floret closing to complete closure, probably due to similar pollination effect. However, it is not clear why the sterile varieties showed reduced levels of IAA and ABA compared with those of fertile lines.

Rice floret opening and closure is proposed to be caused by turgor pressure movement like guard cells [18]. It is well-known that ABA regulates turgor pressure in stomata guard cells, causes water loss in the cells and promote stomata closure in leaves. We therefore speculate that the mechanism of ABA-promoted floret closure is similar to that of the stomata closure. It is generally accepted that auxins increase water uptake and cell expansion. Therefore, floret closing in rice delayed by auxins might be due to auxin effects on promoting water uptake and/or preventing water loss in floret cells. More direct evidence is required to demonstrate the mechanism of rice floret opening and closing by auxin and ABA.

Currently, there is limited knowledge about the mechanism and regulation of floret opening and closing. It is generally believed that floret opening is due to water uptake which causes cell expansion in lodicule. After floret flowering, water loss in lodicule causes shrinkage in the tissue and pushes inferior lemma towards palea and eventually results in the closing of floret. Water loss in lodicule is caused by active export of water and solutes from the tissue to rachilla. The reasons for the slower floret closure in sterile rice are probably due to fewer number of vascular tissues and poorer development of the transportation system [6,27]. After spikelet opening, lodicule is exposed out of inferior lemma and palea. Under microscopy, the outer cell wall of the epidemic cells in lodicule apparently becomes thick, reaches approximately 3–5 fold of thickness of those in the parenchyma cell walls and becomes a waterproof layer. The fact that floret closing is not affected by atmospheric moisture suggests that floret closing is not due to loss of water on the surface of epidermis [6].

No prior research had shown what initiates floret closing. Based on this study, we speculate that when floret opening reaches the maximal angle, the decrease of endogenous IAA levels and increase of endogenous ABA levels initiate the transition from opening to closing of florets. This study provides insights towards better understanding of the regulatory mechanism of floret closing in rice. Because of the stimulation of floret closing by ABA, it is potentially important to increase the yield and improve the quality of hybrid seed by shortening the length of floret opening. It is worth of keeping in mind that this study only studied the endogenous IAA and ABA levels in the whole flowers due to limited availability of locidule tissues. Further study on IAA and ABA content in locidule tissues is needed to illustrate the mechanism of hormonal regulation of floret closing in rice.

## Abbreviations

ABA: abscisic acid
IAA: indole-3-acidic acid
IBA: indole-3-butyric acid
JA: jasmonate
MeJA: methyl jasmonate
NAA: 1-naphthalene-acetic acid
2,4-D: 2,4-dichlorophenoxy acetic acid
DIC: 3,6-dichloro-2-methoxybenzoic acid
ELISA: enzyme-linked immunosorbent assay
SA: salicylic acid
